# Moyamoya Disease Revealed by Ruptured Intraventricular Aneurysm

**DOI:** 10.5334/jbsr.2171

**Published:** 2020-09-09

**Authors:** Katelijn Pannecoeck, Eva Genbrugge, Peter Vanlangenhove

**Affiliations:** 1 UZ Gent, BE

**Keywords:** Moyamoya, aneurysm, neuroradiology, interventional radiology, angiography

## Abstract

**Teaching point:** Narrowing and occlusion of the distal carotids in Moyamoya causes a change in blood flow dynamics, increasing the risk for intracranial aneurysm formation.

## Case

**Figure 1:**
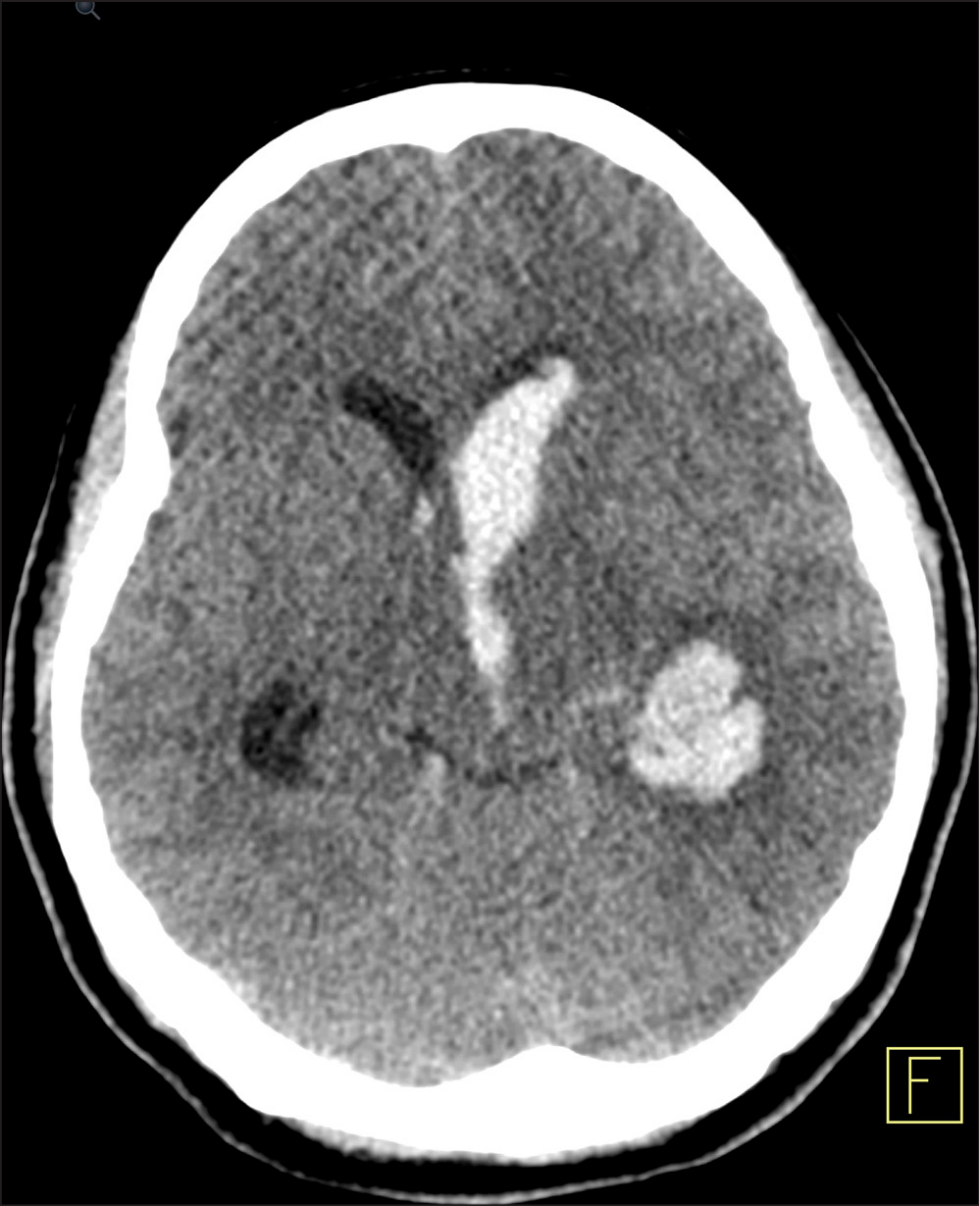


**Figure 2:**
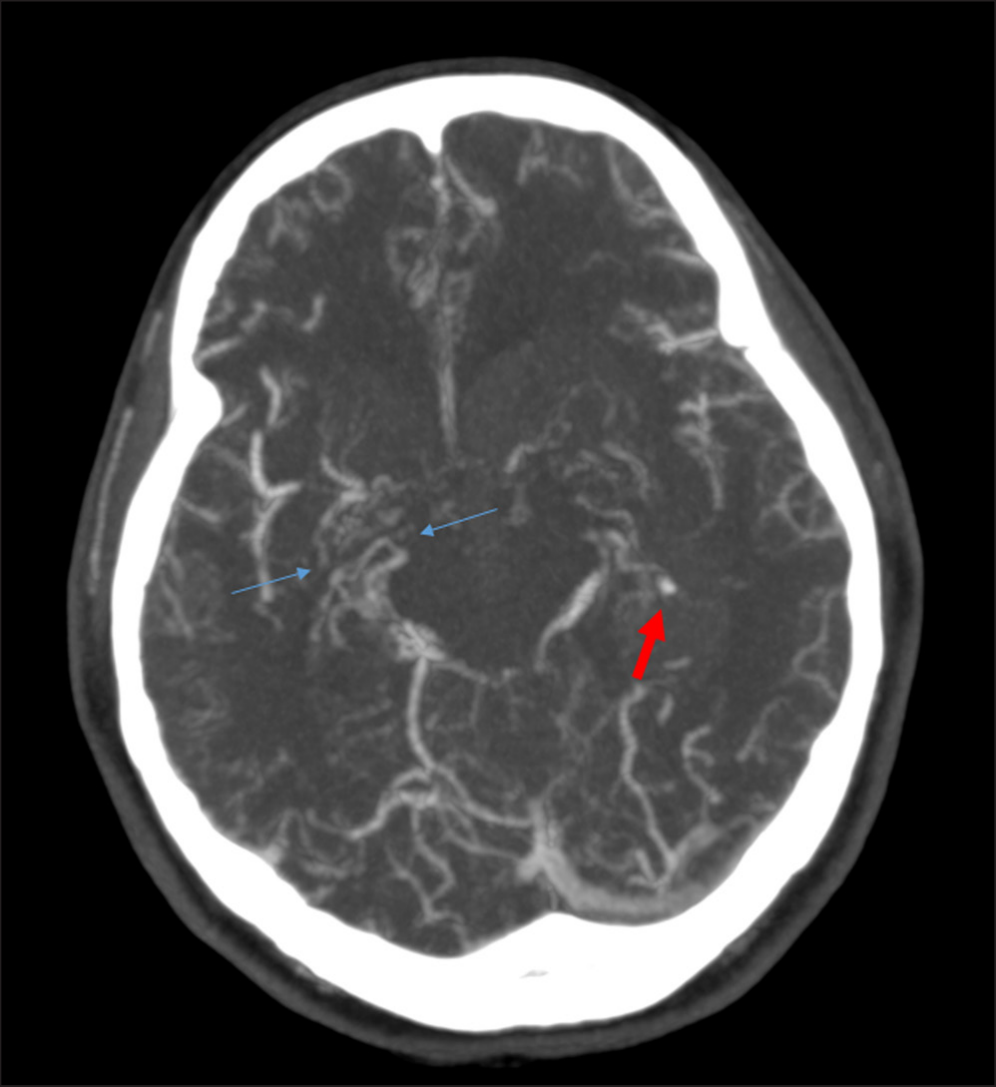


A 28-year-old Korean woman was brought to our emergency department with repeated vomiting after falling down on the street. Physical examination showed no external injury, although there was an impaired neurological function with a Glasgow Coma Score of only 10/15. Blood tests were normal, including toxicology screening. Because of the abnormal neurological exam, a computed tomography (CT) of the brain was performed and showed an extensive intraventricular hemorrhage, with predominance in the left lateral ventricle and slight periventricular hypodensity (edema) (Figure 1). Considering the absence of external injury and the distribution of the intracranial blood, we additionally performed CT angiography that showed a left intraventricular aneurysm at the center of the hemorrhage (Figure 2; red arrow). There were also bilateral caliber irregularities of the distal internal carotids and the proximal middle cerebral arteries, with a severe narrowing on the right side and a complete occlusion on the left side. The anterior cerebral arteries were not clearly depictable on both sides. Multiple small collateral arteries were seen in the basal ganglia (Figure 2; blue arrows).

**Figure 3:**
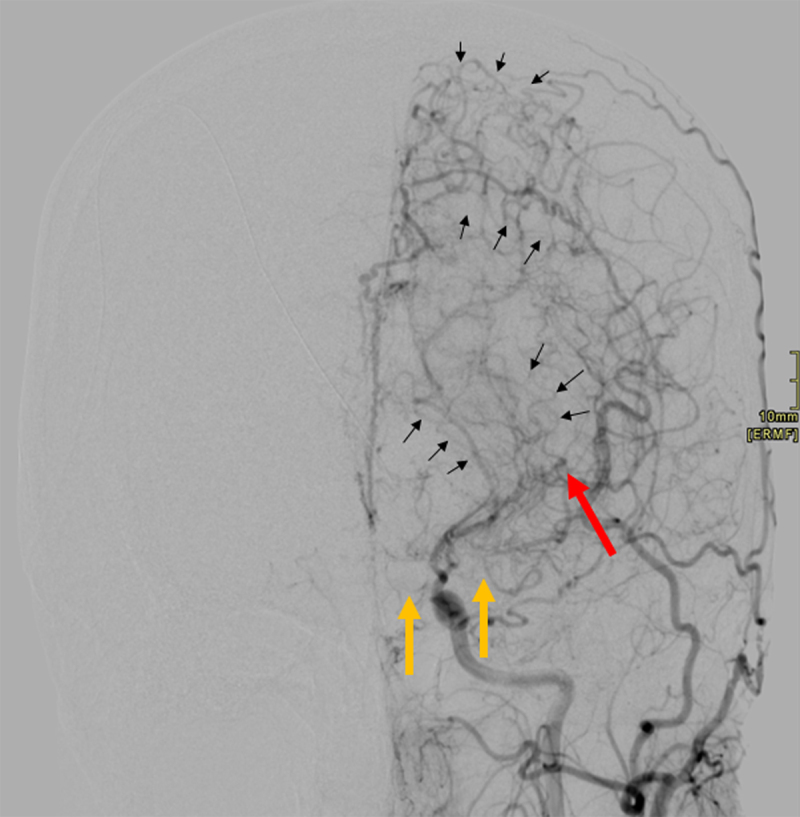


Conventional cerebral arteriography confirmed the initial diagnosis, showing the choroid plexus aneurysm (Figure 3; red arrow), occlusion of both the left anterior and middle cerebral arteries (A1 and M1 segments, Figure 3; yellow arrows) and multiple intracranial collaterals (Figure 3; multiple small black arrows) exhibiting the classic “puff of smoke” dynamic appearance of Moyamoya disease.

## Comment

Moyamoya is a chronic, progressive cerebral vasculopathy affecting the intracranial part of the internal carotid arteries and its main side branches; causing stenosis and/or complete occlusion of the affected vessels. Thus, the main differential diagnosis is vessel narrowing caused by arteriosclerosis, and this should always be excluded.

The disease is most prevalent in the Asian population, especially in Japan, with an incidence of up to 10.5 per 100,000 people and an associated positive family history in up to 12% of patients [1]. Both findings strongly suggest an underlying genetic etiology. The exact pathophysiological processes are still unknown. It occurs in all age groups, with a bimodal peak distribution of the age of onset: a first peak in early childhood (4–10 years old) and a second peak in middle aged patients (30–40 years old) [1]. The clinical manifestations are diverse and mainly represent ischemic stroke, hemorrhage, and epilepsy.

Pathognomonic imaging findings are bilateral, concentric narrowing and/or occlusion of the distal internal carotid arteries and circle of Willis, with subsequent formation of intracranial collaterals, causing the typical “puff of smoke” appearance on the “gold-standard” dynamic catheter angiography. Hemodynamic and wall disease changes both contribute to aneurysm formation, which should not be overlooked on imaging, as present in approximately 14% of adults with Moyamoya disease.
